# Follow-up of Intervention to Prevent Dental Caries Among Indigenous Children in Australia: A Secondary Analysis of a Randomized Clinical Trial

**DOI:** 10.1001/jamanetworkopen.2019.15611

**Published:** 2019-11-27

**Authors:** Lisa Jamieson, Lisa Smithers, Joanne Hedges, Helen Mills, Kostas Kapellas, Diep Ha, Loc Do, Xiangqun Ju

**Affiliations:** 1 Australian Research Centre for Population Oral Health, Adelaide Dental School, University of Adelaide, Adelaide, South Australia, Australia; 2 School of Public Health, University of Adelaide, Adelaide, South Australia, Australia

## Abstract

**Importance:**

For an intervention delivered in early childhood to have meaningful translational effect, long-term follow-up is necessary, especially among underserved indigenous children among whom preventable dental disease is common.

**Objectives:**

To test the long-term effectiveness of an early-childhood dental intervention through a follow-up at age 5 years among Aboriginal children in Australia.

**Design, Setting, and Participants:**

This secondary analysis of a randomized clinical trial followed up on participants of the Baby Teeth Talk Study, a 2-group parallel, outcome assessor–blinded, randomized clinical trial conducted among Aboriginal children in South Australia, Australia. Participants included 448 mother or caregiver–child dyads who were enrolled in the Baby Teeth Talk trial between February 2010 and May 2011 and were randomized in the present trial to the immediate intervention group or the delayed intervention group. Intention-to-treat principles were used for all data analyses to estimate the effect of the intervention on dental caries experience. Data analysis was performed from April 10 to May 27, 2019.

**Interventions:**

The intervention comprised 4 services to participants: (1) dental care to mothers during pregnancy, (2) application of fluoride varnish to children’s teeth, (3) anticipatory guidance in the form of oral health educational packages, and (4) motivational interviewing for pregnant mothers and children at ages 6, 12, and 18 months in the immediate intervention group and at ages 24, 30, and 36 months in the delayed intervention group.

**Main Outcomes and Measures:**

The primary outcome was the mean number, in the primary dentition, of decayed, missing, or filled teeth (dmft) at age 5 years. Individual components of the dmft index were examined, as was the prevalence of dmft greater than 0.

**Results:**

Of the 449 Aboriginal mothers and children recruited, 223 (49.7%) were randomized to the immediate intervention group and 225 (50.1%) to the delayed intervention group. The mean dmft at age 5 years was 2.10 (95% CI, 2.04 to 2.16) for children in the immediate intervention group and 2.91 (95% CI, 2.83 to 3.00) for children in the delayed intervention group (adjusted mean difference, −1.02; 95% CI, −1.81 to −0.22). When considering children in nonmetropolitan locations, the differences were stark; the mean dmft was 2.46 (95% CI, 2.38-2.54) for children in the immediate intervention group and 3.65 (95% CI, 3.53 to 3.78) for children in the delayed intervention group, with an adjusted mean difference of −1.52 (95% CI, −2.61 to −0.43). Most of this difference was accounted for by missing teeth, with the mean number of missing teeth of children in the immediate intervention group living in nonmetropolitan locations being 0.29 (95% CI, 0.27 to 0.31) compared with 1.02 (95% CI, 0.96 to 1.07) for their counterparts in the delayed intervention group. A 3-fold difference was observed in the percentage of missing teeth greater than 0 between children in the immediate intervention group and those in the delayed intervention group (10.8 [95% CI, 10.2 to 11.4] vs 31.0 [95% CI, 30.1 to 31.8]).

**Conclusions and Relevance:**

This trial found that a multifaceted initiative to reduce early-childhood caries continued to be efficacious in participating indigenous children aged 5 years, especially those residing in nonmetropolitan locations and with teeth missing because of dental disease.

**Trial Registration:**

anzctr.org.au Identifier: ACTRN12611000111976

## Key Points

**Question:**

Is an intervention to prevent early-childhood caries still effective at a 5-year follow-up among Aboriginal children in Australia?

**Findings:**

In this secondary analysis of a randomized clinical trial that followed up on 448 mothers or caregivers and children who participated in a trial to prevent childhood dental disease, children who received the dental intervention within 6 to 18 months of life had less experience of dental disease than their counterparts in the delayed intervention group.

**Meaning:**

This trial found evidence that a multipronged, culturally safe intervention to prevent early-childhood caries delivered earlier rather than later in infancy was still efficacious at 5 years of follow-up.

## Teaser

This secondary analysis of a randomized clinical trial investigates the long-term success of a dental health intervention introduced in a trial involving Aboriginal and Torres Strait Islander children and their mothers in Australia.

## Introduction

Determining the long-term efficacy of an intervention implemented in early childhood is important for translation and policy implications. In dentistry, many randomized clinical trials conducted in childhood report the efficacy of a given intervention at the study end, but no long-term follow-up is usually performed. Examples of such studies without follow-up include interventions for behavior change,^1^ application of therapeutic agents,^2^ and psychosocial counseling^3^ and education.^4^ Usually, meta-analyses are required before findings from a given intervention will be recommended for clinician guidelines and/or policy (for example, recommendations for use of topical fluoride varnish^5^). However, the interventions informing these meta-analyses typically do not include long-term follow-up.

In Australia, Aboriginal and Torres Strait Islanders are recognized as the original inhabitants of the country, having thrived for up to 65 000 years before European colonization.^6^ Since that time, the health and welfare of Aboriginal and Torres Strait Islander Australians has dramatically decreased, largely owing to the introduction of infectious diseases; processed foods; and sustained government policies of land dispossession, economic and social discrimination, and removal of children from families.^7^ Aboriginal children in Australia have poorer early life development outcomes compared with non-Aboriginal children,^8^ as well as lower educational attainment. Evidence from a recent national report on the Australian Early Development Census, which included data on almost 290 000 Australian children aged 5 years, demonstrated that approximately half of the Aboriginal children were susceptible on 1 or more of the physical, social, emotional, cognitive or language, and communication domains. This number was more than twice the percentage reported for non-Aboriginal children.^9^ Experience of preventable dental disease is also high among Aboriginal children in Australia. In the 2012-2014 National Child Oral Health Study (NCOHS), the mean number of decayed, missing, or filled tooth surfaces in the primary dentition of Aboriginal and Torres Strait Islander children aged 5 to 10 years was 6.3, compared with 2.9 among non–Aboriginal and Torres Strait Islander children.^10^

The Baby Teeth Talk Study (a name provided by the trial’s Aboriginal Reference Group) was an early-childhood caries intervention conducted among Aboriginal children and their families in South Australia. The trial proved to be efficacious, with children in the immediate intervention group having less untreated dental caries than children in the delayed intervention group at follow-up conducted at 2 and 3 years of age.^11,12^

In this secondary analysis of a randomized clinical trial, we tested the efficacy of the Baby Teeth Talk Study intervention when participating children reached the age of 5 years. Our first hypothesis was that, among the immediate intervention group, the efficacy of the intervention would persist at age 5 years. We also investigated levels of dental disease experience in the whole sample compared with national-level estimates. Our next hypotheses were that (2) children in the Baby Teeth Talk Study at age 5 years would have higher levels of dental disease than children in the 2012-2014 NCOHS and (3) children in the Baby Teeth Talk trial at age 5 years would have lower levels of dental disease than their Aboriginal counterparts in the NCOHS.

## Methods

### Background

This secondary analaysis was a 5-year follow-up of the Baby Teeth Talk Study, a 2-group parallel, outcome assessor–blinded, randomized clinical trial conducted in South Australia, Australia.^13^ As a follow-up study, this work received ethical approval from the University of Adelaide Human Research Ethics Committee, the Aboriginal Health Council of South Australia, the Government of South Australia, and the Human Research Ethics Committees of participating South Australian hospitals. Informed consent was not required for this trial because written informed consent was provided by all participants during the original study. We followed the Consolidated Standards of Reporting Trials (CONSORT) reporting guideline.

The primary outcome of the current study was the mean number of decayed, missing, or filled teeth (dmft) in the primary dentition of children at 5 years of age. Individual components of the dmft index were examined, as was prevalence of dmft higher than 0. Data from the 2012-2014 NCOHS^10^ were used as a comparator for hypotheses 2 and 3.

### Participants and Follow-Up

Interested women who self-identified as pregnant with an Aboriginal and/or Torres Strait Islander child were enrolled in the Baby Teeth Talk trial between February 2010 and May 2011. The final sample was representative of the population by maternal age, socioeconomic status, and tobacco smoking status and comprised two-thirds of the total number of women pregnant with Aboriginal and Torres Strait Islander children during the recruitment period.^14^ Recruitment was through the antenatal clinics of South Australian Aboriginal Community Controlled Health Organisations and hospitals. All participants provided written informed consent. Good Clinical Practice guidelines and the ethical standards of the Declaration of Helsinki^15^ were followed for the Baby Teeth Talk Study and subsequent follow-up. The University of Adelaide Human Research Ethics Committee, the Aboriginal Health Council of South Australia, the Government of South Australia, and the Human Research Ethics Committees of participating South Australian hospitals provided ethical approval.

We randomized 449 women to either the immediate intervention group or delayed intervention group (Figure), and participants were provided with a number of services. First, mothers who were allocated randomly to the immediate intervention group and who were eligible for publicly funded dental care (through ownership of a means-tested government health care card) received dental care through the South Australian Dental Service. Study staff organized transport and appointments with assistance from the South Australian Dental Service Aboriginal Liaison Program. Six private dentists provided care to participants who were not eligible for publicly funded care. Dental care included x-rays, checkups, scale and prophylaxis, fillings, and extractions (including wisdom teeth). Not provided were cosmetic dentistry, endodontics, and orthodontics. Second, fluoride varnish was applied for children at ages 6, 12, and 18 months. The protocol for fluoride varnish was based on that used by Slade and colleagues.^16^ Study staff were trained to apply the varnish. Children were supine, and their teeth were cleaned and dried with gauze. Fluoride varnish was applied from the back teeth forward to the front teeth. Mothers or caregivers for the children were advised to not give or allow the children to consume food or drink for half an hour.

**Figure.**
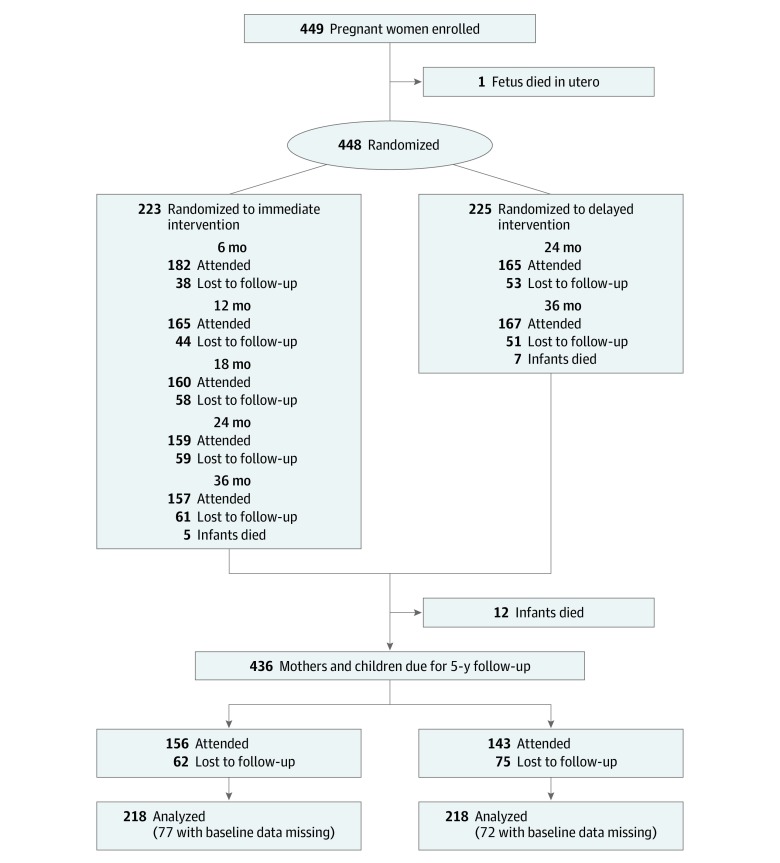
Flow Diagram of Participants Immediate intervention included (1) dental care during pregnancy, (2) anticipatory guidance for mother, (3) motivational interviewing for mother, and (4) fluoride varnish application for child. Delayed intervention included (1) anticipatory guidance for mother, (2) motivational interviewing for mother, and (3) fluoride varnish application for child.

Third, participants received anticipatory guidance in the form of oral health educational packages that contained dental information relevant for pregnant mothers (ie, focus on dental treatment and pregnancy gingivitis) and children aged 6 months (ie, focus on first solids and caring for baby teeth on initial eruption), 12 months (ie, focus on tooth brushing and fluoride use and avoiding sugar-containing beverages and foods), and 18 months (ie, focus on child’s first dental checkup and molar teeth eruption). Fourth, in combination with anticipatory guidance, motivational interviewing was implemented for pregnant mothers and children at ages 6, 12, and 18 months. Study staff completed an initial 2-day motivational interviewing training course followed by an intensive 1-day follow-up that continued monthly for 6 months. One-day coaching was also provided every 2 months, followed by occasional telephone coaching for 1 full year. Each motivational interviewing session was conducted on a one-to-one basis in venues where participants felt comfortable (for example, community halls, local Aboriginal health services, and participants’ homes). Motivational interviewing sessions ranged from 30 to 90 minutes. Pictorial prompts and plain English summaries were used.^17^ A member of the Motivational Interviewing Network of Trainers conducted the fidelity testing of the motivational interviewing sessions, which were found to be acceptable.^18^

In the delayed intervention group, mothers received dental care when children were aged 24 months. Fluoride varnish application was delivered to children at age 24 months, anticipatory guidance was delivered at age 30 months, and motivational interviewing was conducted at age 36 months.

When children in both groups were followed up at ages 24, 36, and 60 months, blinded and calibrated examiners conducted oral examinations. A standardized protocol to record dental disease experience was followed. Teeth were dried with cotton pads before examination. A fiber-optic light was used as a light source, and standard infection control procedures were followed. Only visual criteria were used for diagnosis, with measurement including the dmft index (d, untreated dental caries; m, teeth missing owing to dental disease experience; and f, teeth filled owing to dental disease experience). Three examiners were tested in the field against a criterion standard examiner to estimate interexaminer reliability. Intraclass correlation coefficients for caries assessment scores ranged from 0.80 to 0.88, indicating good to excellent reliability. Children were referred to the South Australian Dental Service if a diagnosis of untreated dental caries was recorded.

The NCOHS was a cross-sectional, population-based survey of children in Australia aged 5 to 14 years.^10^ The NCOHS used a 2-stage stratified sample design to ensure a representative sample of children from across Australia. In the first stage, a sampling school was created from a list provided by each state and territory. This sampling included public, Catholic, and independent primary and secondary schools. Schools were then selected with a probability proportional to the size of enrollment. In the second stage, a child cluster was randomly sampled from each participating school. Mothers or caregivers of selected children were requested to participate, and participation included completion of a parental or caregiver questionnaire and a child oral epidemiological examination.

Ethics approval for the NCOHS was obtained from the University of Adelaide Human Research Ethics Committee, research ethics committees within each jurisdiction, the 3 education sectors (public, independent, and Catholic schools), and the Indigenous Human Research Ethics Committee at the Menzies Institute. Mothers or caregivers signed an informed consent for their child to participate. Child data from each state and territory were weighted separately, with survey weights adjusted by school type and child sociodemographic characteristics. The weighted sample’s population estimates closely matched those of the true child population.^10^ For purposes of this comparative analysis, data for only children aged 5 years were used.

### Outcomes and Descriptive Data

The primary outcome was the mean number of dmft in the child at a mean age of 5 years. Dental decay was computed at the threshold of both precavitation (an area of demineralization without loss of surface continuity) and cavitation (a visible break in the enamel caused by demineralization). We also examined the prevalence of untreated dental dmft (that is, the proportion of children with 1 or more decayed teeth, 1 or more missing teeth, and 1 or more filled teeth). Descriptive variables at baseline included maternal age, educational level, income, means-tested health care card status, residential location, dental behaviors (usual reason for visiting a dentist, maternal tooth brushing behavior), and health status (self-rated oral health and self-rated general health).

Maternal age was categorized as 14 to 24 years or 25 years or older, and educational level was categorized as high school or less or trade or technical or university degree. Income was dichotomized as job or Centrelink (Australian welfare) and ownership of a means-tested government health care card (yes or no). Centrelink is the Australian government agency that provides welfare payments to those who are eligible (means-tested). Residential location was categorized into metropolitan (Adelaide and outer suburbs) and nonmetropolitan (regional areas).

Dental behaviors included the questions, “What is your usual reason for seeing a dentist?” (with response options of problem or check-up) and “Did you brush your teeth yesterday?” (with response options of yes or no). Mothers’ or caregivers’ self-rated oral and general health status was obtained the question, “How do you think your general/dental health is?” (with response options of, excellent, very good, good, fair, or poor).

### Statistical Analysis

Intention-to-treat principles were used for all data analyses to estimate the effect of the intervention on dental caries experience. General linear regression models were used to compare the efficacy of the intervention on mean dmft between immediate and delayed intervention groups at age 5 years. To account for any contributing factors, we adjusted for baseline maternal sociodemographic, health status, and dental behavior characteristics. The Proc genmod function was used in SAS, with link=identity and distribution=normal, so the general linear regression models could be fitted and the least squares estimates obtained. To test for hypothesis 1, we calculated the difference in outcome (mean dmft) at age 5 years between the immediate and delayed groups after adjusting for covariates. Because of the differences in Aboriginal child experience of dental caries by residential location,^19^ we decided a priori to also investigate efficacy by residential location.

Because by child age of 5 years nearly one-third of mother-child pairs were lost to follow-up, a fully conditional specification method was used to impute missing data based on the assumption that data were missing at random. Immediate and delayed intervention groups were imputed separately. Fifty imputed data sets were created using 50 iterations, with the results from the imputed data sets combined using Rubin’s rules via the Proc mianalyse function. Sensitivity analyses were conducted using the MNAR (missing not at random) adjust statement with different scenarios for dental outcomes, which included different percentages of missing-at-random assumptions and maximum and minimum value imputations.

To test for hypotheses 2 and 3, we used basic descriptive statistics to compare 5-year-old findings of the Baby Teeth Talk Study with age-matched estimates in the NCOHS (both total child population and indigenous children only). Differences were considered to be statistically significant when 95% CIs were nonoverlapping.

SAS, version 9.4 (SAS Institute Inc) was used for all analyses and imputations. Data analysis was performed from April 10 to May 27, 2019.

## Results

We recruited 449 mothers who were pregnant with an Aboriginal child between February 2011 and May 2012. Of the 449 recruited, 223 (49.7%) were randomized to the immediate intervention group and 225 (50.1%) to the delayed intervention group (Figure). One baby died in utero. At baseline, the sociodemographic, dental behavior, and health status characteristics of the 2 intervention groups were similar (Table 1).

**Table 1.** Baseline Maternal Sociodemographic and Dental Behavioral Characteristics

Variable	Baseline No. (%)		
	Total	Immediate Intervention	Delayed Intervention
Total	448	223	225
Maternal age, y			
14-24	283 (53.1)	130 (58.3)	108 (48.0)
≥25	210 (46.9)	93 (41.7)	117 (52.0)
Educational level			
≤High school	322 (72.4)	162 (73.3)	160 (71.4)
Trade or university degree	123 (27.6)	59 (26.7)	64 (28.6)
Income			
Job	62 (14.0)	32 (14.5)	30 (13.5)
Centrelink	381 (86.0)	189 (85.5)	192 (86.5)
HCC status			
Yes	358 (82.2)	175 (81.8)	183 (82.8)
No	77 (17.8)	39 (18.2)	38 (17.3)
Residential location			
Metropolitan	171 (38.7)	79 (35.9)	92 (41.4)
Nonmetropolitan	271 (61.3)	141 (64.1)	130 58.6)
Usual reason for dentist visit			
Problem	275 (64.0)	141 (65.0)	134 (62.9)
Checkup	155 (36.1)	76 (35.0)	79 (37.1)
Brush yesterday			
Yes	321 (75.0)	158 (74.2)	163 (75.8)
No	107 (25.0)	55 (25.8)	52 (24.2)
Self-rated oral health			
Excellent, very good, good	203 (45.3)	90 (40.4)	113 (50.2)
Fair or poor	245 (54.7)	133 (59.6)	112 (49.8)
Self-rated general health			
Excellent, very good, good	402 (89.9)	197 (88.7)	205 (91.1)
Fair or poor	45 (10.1)	25 (11.3)	20 (8.9)

Abbreviation: HCC, health care card.

The first follow-up visit was conducted at child mean age of 2 years, with clinical dental data available for 324 children (159 in the immediate intervention group and 165 in the delayed intervention group). The second follow-up visit was conducted at child mean age of 3 years, with clinical dental data available for 324 children (157 in the immediate intervention group and 167 in the delayed intervention group). At the third follow-up, when children were aged 5 years, clinical data were available for 156 children in the immediate intervention group and 143 in the delayed intervention group (total of 299). Maternal baseline characteristics were mostly similar for children lost to follow-up at 5 years of age compared with those who remained in the study (eTable in the Supplement). The exceptions were usual reason for dental visit (63.8% [n = 83] for a problem and 36.2% [n = 47] for a check-up; *P* = .03) and self-rated oral health (48.2% [n = 66] excellent, very good, or good and 51.8% [n = 71] fair or poor; *P* = .02) for children lost to follow-up at age 5 years.

Table 2 presents dental caries experience at child age of 5 years. The mean number of dmft was 2.10 (95% CI, 2.04 to 2.16) for the immediate intervention group and 2.91 (95% CI, 2.83 to 3.00) for the delayed intervention group, with an adjusted mean difference of −1.02 (95% CI, −1.81 to −0.22). This finding means that children in the delayed intervention group had a mean of 1 more whole tooth that had experienced dental disease than their counterparts in the immediate intervention group had. The preventive fraction from the adjusted model was 35.1%. Statistically significant differences were observed in the missing teeth estimates, with the mean missing teeth being 0.31 (95% CI, 0.29 to 0.34) for children in the immediate intervention group and 0.79 (95% CI, 0.75 to 0.83) for children in the delayed intervention. The adjusted mean difference was −0.48 (95% CI, −0.81 to −0.15), with a preventive fraction of 60.8%. Metropolitan-dwelling children had significantly less experience of dental disease than nonmetropolitan-dwelling children at age 5 years, irrespective of intervention group. When considering children in nonmetropolitan locations, the mean dmft for children in the immediate intervention group was 2.46 (95% CI, 2.38 to 2.54) and for children in the delayed intervention was 3.65 (95% CI, 3.53 to 3.78), with an adjusted mean difference of −1.52 (95% CI, −2.61 to −0.43); that is, children in the delayed intervention group living in nonmetropolitan areas had 1.5 more teeth affected by dental disease than children in the immediate intervention group living in nonmetropolitan areas. The preventive fraction was 17.6%.

**Table 2.** Experience of Dental Disease at Child Age 5 Years by Intervention Group and Residential Location

Variable	Mean (95% CI)		Unadjusted		Adjusted^a^	
	Immediate Intervention	Delayed Intervention	Mean Difference (95% CI)^b^	Prevented Fraction, %	Mean Difference (95% CI)^b^	Prevented Fraction, %
Severity of dental disease						
Mean dt	1.30 (1.25 to 1.34)	1.61 (1.54 to 1.66)	−0.31 (−0.88 to 0.28)	18.8	−0.48 (−1.05 to 0.09)	30.0
Mean mt	0.31 (0.29 to 0.34)	0.79 (0.75 − 0.83)	−0.47 (−0.80 to −0.14)	59.5	−0.48 (−0.81 to −0.15)	60.8
Mean ft	0.49 (0.46 to 0.51)	0.50 (0.48 to 0.53)	−0.01 (−0.30 to 0.27)	2.0	−0.04 (−0.33 to 0.24)	8.0
Mean dmft	2.10 (2.04 to 2.16)	2.91 (2.83 to 3.00)	−0.81 (−1.62 to −0.00)	27.8	−1.02 (−1.81 to −0.22)	35.1
Mean dt by residential location						
Metropolitan	0.79 (0.74 to 0.84)	1.16 (1.09 to 1.23)	−0.37 (−1.11 to 0.37)	31.9	−0.37 (−1.10 to 0.37)	31.9
Nonmetropolitan	1.59 (1.52 to 1.65)	1.91 (1.83 to 2.00)	−0.33 (−1.14 to 0.48)	17.3	−0.64 (−1.45 to 0.17)	33.5
Mean mt by residential location						
Metropolitan	0.37 (0.32 to 0.42)	0.47 (0.42 to 0.51)	−0.10 (−0.60 to 0.41)	21.3	0.15 (−0.30 to 0.61)	31.9
Nonmetropolitan	0.29 (0.27 to 0.31)	1.02 (0.96 to 1.07)	−0.73 (−1.17 to −0.30)	71.6	−0.39 (0.76 to −0.03)	38.2
Mean ft by residential location						
Metropolitan	0.35 (0.32 to 0.39)	0.30 (0.27 to 0.34)	0.05 (−0.31 to 0.41)	16.7	0.13 (−0.21 to 0.47)	43.3
Nonmetropolitan	0.57 (0.53 to 0.60)	0.64 (0.60 to 0.68)	−0.08 (−0.47 to 0.31)	12.5	0.12 (−0.27 to 0.51)	18.8
Mean dmft by residential location						
Metropolitan	1.48 (1.39 to 1.56)	1.88 (1.77 to 1.98)	−0.40 (−1.50 to 0.70)	21.3	−0.33 (−1.41 to 0.76)	21.3
Nonmetropolitan	2.46 (2.38 to 2.54)	3.65 (3.53 to 3.78)	−1.19 (−2.28 to −0.11)	32.6	−1.52 (−2.61 to −0.43)	17.6
Prevalence of dental disease						
% dt >0	36.4 (35.5 to 37.3)	35.2 (34.3 to 36.0)	1.3 (0.0 to 2.6)	3.6	−0.9 (−2.2 to 0.4)	2.5
% mt >0	10.8 (10.2 to 11.4)	31.0 (30.1 to 31.8)	−20.2 (−21.2 to −19.1)	65.2	−21.4 (−22.4 to −20.4)	69.1
% ft >0	19.2 (18.4 to 19.9)	19.9 (19.1 to 20.6)	−7.3 (−1.8 to 0.3)	36.9	−1.2 (−2.8 to −0.2)	6.1
% dmft >0	46.3 (45.4 to 47.3)	45.1 (44.1 to 46.0)	1.3 (−0.1 to 2.6)	2.8	−0.5 (−1.8 to 0.8)	1.0

Abbreviations: dmft, decayed, missing, or filled teeth; dt, decayed teeth; ft, filled teeth; mt, missing teeth because of dental disease.

^a^ Adjusted for baseline maternal sociodemographic, health status, and dental behavior characteristics.

^b^ For the prevalence of dental disease subcategories, data are presented as risk difference in proportion (95% CI).

Stark differences were found when missing teeth were considered in isolation, with the mean missing teeth of children in the immediate intervention group in nonmetropolitan locations being 0.29 (95% CI, 0.27 to 0.31), compared with 1.02 (95% CI, 0.96 to 1.07) for children in the delayed intervention group in these areas. For children in the delayed intervention group residing in nonmetropolitan areas, the missing teeth component was greater than the filled teeth component (that is, more children had teeth extracted than teeth filled). When considering the prevalence of missing teeth, we found that the percentage of missing teeth greater than 0 for children in the immediate intervention group was 10.8 (95% CI, 10.2 to 11.4), compared with 31.0 (95% CI, 30.1 to 31.8) for children in the delayed intervention group, a 3-fold difference. The adjusted risk difference in proportion was −21.4 (95% CI, −22.4 to −20.4), with a preventive fraction of 69.1%.

When compared with population estimates from the 2012-2014 NCOHS (Table 3), 5-year-old participants in the Baby Teeth Talk Study had profoundly higher levels of dental disease irrespective of indicator used. Baby Teeth Talk, compared with NCOHS, participants had 1.7 times the mean number of decayed teeth (1.42 [95% CI, 1.38-1.44] vs 0.82 [95% CI, 0.74-0.91]), 7.9 times the mean number of missing teeth (0.55 [95% CI, 0.53-0.58] vs 0.07 [95% CI, 0.04-0.09]), 1.4 times the mean number of filled teeth (0.49 [95% CI, 0.48-0.51] vs 0.34 [95% CI, 0.29-0.40]), and 2.0 times overall dmft (2.47 [95% CI, 2.42-2.52] vs 1.23 [95% CI, 1.12-1.34]). Similar findings were observed when prevalence of dental disease was considered, with Baby Teeth Talk participants having 1.4 times the percentage of decayed teeth greater than 0 (34.9 [95% CI, 34.2-35.5] vs 25.0 [95% CI, 22.4-27.7]), 7.7 times the percentage of missing teeth greater than 0 (20.9 [95% CI, 20.3-21.4] vs 2.7 [95% CI, 1.7-3.6]), 1.6 times the percentage of filled teeth greater than 0 (19.5 [95% CI, 19.0-20.0] vs 12.2 [95% CI, 10.2-14.2]), and 1.5 times the overall percentage of dmft greater than 0 (46.5 [95% CI, 45.8-47.2] vs 31.0 [95% CI, 28.2-33.8]).

**Table 3.** Comparing Dental Caries Experience Among Australian Children Aged 5 Years

Measure	Mean (95% CI)		
	BTT Study (MI)	NCOHS (Weighted)	
	Indigenous (n = 436)	All (n = 2154)	Indigenous (n = 94)
dt	1.42 (1.38-1.44)	0.82 (0.74-0.91)	1.83 (1.16-2.51)
mt	0.55 (0.53-0.58)	0.07 (0.04-0.09)	0.35 (0.02-0.69)
ft	0.49 (0.48-0.51)	0.34 (0.29-0.40)	0.44 (0.23-0.65)
dmft	2.47 (2.42-2.52)	1.23 (1.12-1.34)	2.62 (1.82-3.44)
Prevalence			
% dt >0	34.9 (34.2-35.5)	25.0 (22.4-27.7)	42.7 (29.2-56.10)
% mt >0	20.9 (20.3-21.4)	2.7 (1.7-3.6)	5.5 (2.9-11.3)
% ft >0	19.5 (19.0-20.0)	12.2 (10.2-14.2)	21.6 (10.0-33.2)
% dmft >0	46.5 (45.8-47.2)	31.0 (28.2-33.8)	47.3 (33.5-61.0)

Abbreviations: BTT, Baby Teeth Talk (the BTT sample had a mixture of immediate treatment and delayed intervention); dmft, decayed, missing, or filled teeth; dt, decayed teeth; ft, filled teeth; MI, multiple imputation; mt, missing teeth; NCOHS, National Child Oral Health Study 2012-2014.

Although Baby Teeth Talk participants, compared with indigenous counterparts from the NCOHS, aged 5 years had fewer mean decayed teeth (1.42 [95% CI, 1.38-1.44] vs 1.83 [95% CI, 1.16-2.51]), fewer mean dmft (2.47 [95% CI, 2.42-2.52] vs 2.62 [95% CI, 1.82-3.44]), smaller percentage of decayed teeth greater than 0 (34.9 [95% CI, 34.2-35.5] vs 42.7 [95% CI, 29.2-56.10]), smaller percentage of filled teeth greater than 0 (19.5 [95% CI, 19.0-20.0] vs 21.6 [95% CI, 10.0-33.2]), and smaller percentage of dmft greater than 0 (46.5 [95% CI, 45.8-47.2] vs 47.3 [95% CI, 33.5-61.0]), the differences were not statistically significant in terms of nonoverlapping 95% CIs (possibly owing to the small sample size of Aboriginal children in the NCOHS). The only statistically significant difference observed was for missing teeth, with Baby Teeth Talk participants having 3.8 times the percentage of missing teeth greater than 0 over their 5-year-old indigenous counterparts in the NCOHS (20.9 [95% CI, 20.3-21.4] vs 5.5 [95% CI, 2.9-11.3]).

## Discussion

In this secondary analysis of a randomized clinical trial, we tested the hypotheses that (1) efficacy of a culturally informed, multipronged early-childhood caries intervention delivered to Aboriginal children and their mothers or caregivers would be demonstrated at the follow-up when the child reached 5 years of age, (2) child participants in the Baby Teeth Talk Study would have higher levels of dental disease than participants in the 2012-2014 NCOHS, and (3) Baby Teeth Talk Study participants would have lower levels of dental disease than the nationally representative indigenous population in the NCOHS.

The findings appear to support hypotheses 1 and 2. At the follow-up age of 5 years, children who received the intervention in utero and into early infancy had less clinically detected dmft than their counterparts in the delayed intervention group. The intervention was especially efficacious for children residing in nonmetropolitan locations and who were missing owing to dental disease (hypothesis 1). Five-year-old Baby Teeth Talk participants had profoundly greater experience of dental disease prevalence and severity than 5-year-old children in the NCOHS, particularly with regard to missing teeth (hypothesis 2). However, hypothesis 3 did not hold, as no statistically significant differences were observed in oral health outcomes between Baby Teeth Talk participants and Aboriginal children in the NCOHS, with the exception of missing teeth (Baby Teeth Talk participants had more missing teeth).

With respect to Baby Teeth Talk participants in the delayed intervention group, it is concerning that such a large proportion of children residing in nonmetropolitan areas had missing teeth that had been extracted owing to dental decay, as opposed to teeth that had been filled. Evidence suggests that if a child presents with extensive dental disease in a nonmetropolitan location, where dental services may be less available than in urban centers, decisions may be based on the availability of emergency care should restorative treatment prove unsuccessful (for example, filling resulting in abscess).^20^ In these instances, the oral health practitioner may make the decision that an extraction is a better treatment option because it ensures no follow-up complications even when, diagnostically, a restoration may be the optimal outcome. In addition, the interaction between risk factors, such as lower educational level and household income of the mother or caregiver,^21^ limited access to fresh food produce, and lack of toothbrush and fluoridated toothpaste^22^ in nonmetropolitan locations, may result in increased levels of dental disease.

Likewise, it is concerning that Baby Teeth Talk participants overall had such high levels of teeth missing owing to dental disease compared with population estimates, even indigenous population estimates, and despite the small number of indigenous children in the NCOHS (meaning wide 95% CIs). This finding suggests that the dental service delivery model in South Australia may inherently favor extraction over other more rehabilitative services for Aboriginal children.

Although these findings suggest the long-term efficacy of an early-childhood caries initiative delivered early rather than later in infancy, continuing to follow-up with these cohorts is important to assess how the Baby Teeth Talk Study intervention might affect permanent dentition and how the participants might track over time compared with population estimates. Permanent teeth begin to emerge from age 6 years. If the benefits of the intervention might be demonstrated well into middle childhood and with permanent dentition, greater traction may be achieved in implementing policy translation and clinical guidelines at a broader level of a multifaceted, culturally responsive health promotion initiative. In addition, greater benefits may be gained by indigenous children in other parts of the world, many of whom also experience high levels of preventable dental disease.

### Limitations

This study has limitations. It is inevitable to incur missing values because of loss to follow-up. As a result, there may be some bias in the estimation of the amount of dental caries experienced. However, multiple imputation was used to impute all missing cases under each possible scenario, which minimizes biases and allows for a sufficient estimate of the intervention association.

## Conclusion

This secondary analysis of a randomized clinical trial found that an early-childhood caries intervention delivered earlier rather than later in infancy was efficacious at the 5-year follow-up. Participants in the Baby Teeth Talk Study, however, had far greater levels of dental disease than other 5-year-old children at the national level in Australia and had far greater levels of missing teeth than Aboriginal children in the national survey.

## Notes

Supplement. **eTable.** Baseline Sample Characteristics by Follow-up and Loss-to-Follow-up at 5-Year Examinations

## References

[1] Lumsden C, Wolf R, Contento I, Feasibility, acceptability, and short-term behavioral impact of the MySmileBuddy Intervention for early childhood caries. J Health Care Poor Underserved. 2019;30(1):-. doi:10.1353/hpu.2019.0007 30827969

[2] Pukallus ML, Plonka KA, Barnett AG, Walsh LJ, Holcombe TF, Seow WK A randomised, controlled clinical trial comparing chlorhexidine gel and low-dose fluoride toothpaste to prevent early childhood caries. Int J Paediatr Dent. 2013;23(3):216-224. doi:10.1111/j.1365-263X.2012.01248.x 22713081

[3] Batliner TS, Tiwari T, Henderson WG, Randomized trial of motivational interviewing to prevent early childhood caries in American Indian children. JDR Clin Trans Res. 2018;3(4):366-375. doi:10.1177/2380084418787785 30238061 PMC6139581

[4] Muhoozi GKM, Atukunda P, Skaare AB, Effects of nutrition and hygiene education on oral health and growth among toddlers in rural Uganda: follow-up of a cluster-randomised controlled trial. Trop Med Int Health. 2018;23(4):391-404. doi:10.1111/tmi.13036 29381827

[5] Marinho VC, Worthington HV, Walsh T, Clarkson JE Fluoride varnishes for preventing dental caries in children and adolescents. Cochrane Database Syst Rev. 2013;(7):CD002279. doi:10.1002/14651858.CD002279.pub2 23846772 PMC10758998

[6] Lourandos H *Continent of Hunter-Gatherers: New Perspectives in Australian Prehistory* Cambridge, UK: Cambridge University Press; 1997.

[7] The Lancet Closing the gap for Aboriginal health. Lancet. 2019;393(10173):718. doi:10.1016/S0140-6736(19)30405-2 30799002

[8] McEwen EC, Boulton TJ, Smith R Can the gap in Aboriginal outcomes be explained by DOHaD. J Dev Orig Health Dis. 2019;10(1):5-16. doi:10.1017/S2040174418001125 30722808

[9] Australian Government Department of Education Chapter 3: early childhood education and care. In *Report on Government Services 2019* Canberra, Australia: Australian Government Department of Education. https://www.pc.gov.au/research/ongoing/report-on-government-services/2019/child-care-education-and-training/early-childhood-education-and-care/rogs-2019-partb-chapter3.pdf. Accessed May 20, 2019.

[10] Do LG, Spencer AJ, eds. Oral Health of Australian Children: The National Child Oral Health Study 2012–14. Adelaide, Australia: University of Adelaide Press; 2016 https://www.adelaide.edu.au/press/titles/ncohs. Accessed May 20, 2019. doi:10.20851/ncohs

[11] Jamieson L, Smithers L, Hedges J, Dental disease outcomes following a 2-year oral health promotion program for Australian Aboriginal children and their families: a 2-arm parallel, single blind, randomised controlled trial. EClinicalMedicine. 2018;1:43-50. doi:10.1016/j.eclinm.2018.05.001 31193658 PMC6537568

[12] Jamieson LM, Smithers LG, Hedges J, Follow-up of an intervention to reduce dental caries in indigenous Australian children: a secondary analysis of a randomized clinical trial. JAMA Netw Open. 2019;2(3):e190648. doi:10.1001/jamanetworkopen.2019.0648 30874781 PMC6484654

[13] Merrick J, Chong A, Parker E, Reducing disease burden and health inequalities arising from chronic disease among indigenous children: an early childhood caries intervention. BMC Public Health. 2012;12:323. doi:10.1186/1471-2458-12-323 22551058 PMC3413605

[14] Scheil W, Scott J, Catcheside B, Sage L, Kennare R *Pregnancy Outcome in South Australia 2012* Adelaide, Australia: Pregnancy Outcome (Statistics) Unit; 2014 https://www.sahealth.sa.gov.au/wps/wcm/connect/4301aa8048429ccba831e9d5ae66e927/14134.1-Pregnancy+Outcomes+Report%20ONLINESecurev2.pdf?MOD=AJPERES&CACHEID=ROOTWORKSPACE-4301aa8048429ccba831e9d5ae66e927-mMA0eY1. Accessed May 20, 2019.

[15] World Medical Association World Medical Association Declaration of Helsinki: ethical principles for medical research involving human subjects. JAMA. 2013;310(20):2191-2194. doi:10.1001/jama.2013.281053 24141714

[16] Slade GD, Bailie RS, Roberts-Thomson K, Effect of health promotion and fluoride varnish on dental caries among Australian Aboriginal children: results from a community-randomized controlled trial. Community Dent Oral Epidemiol. 2011;39(1):29-43. doi:10.1111/j.1600-0528.2010.00561.x 20707872 PMC3040293

[17] Venner KL, Feldstein SW, Tafoya N Native American Motivational Interviewing: Weaving Native American and Western Practices. Albuquerque, NM: University of New Mexico, Center on Alcoholism, Substance Abuse and Addictions, Department of Psychology; 2006 https://www.integration.samhsa.gov/clinical-practice/Native_American_MI_Manual.pdf. Accessed May 28, 2019.

[18] Jamieson L, Bradshaw J, Lawrence H, Fidelity of motivational interviewing in an early childhood caries intervention involving Indigenous Australian mothers. J Health Care Poor Underserved. 2016;27(1)(suppl):125-138. doi:10.1353/hpu.2016.0036 26853206

[19] Jamieson LM, Armfield JM, Roberts-Thomson KF The role of location in indigenous and non-indigenous child oral health. J Public Health Dent. 2006;66(2):123-130. doi:10.1111/j.1752-7325.2006.tb02567.x 16711632

[20] Jamieson LM, Roberts-Thomson KF Dental general anaesthetic trends among Australian children. BMC Oral Health. 2006;6:16. doi:10.1186/1472-6831-6-16 17184552 PMC1770909

[21] Australian Institute of Health and Welfare. *Australia’s Welfare 2017* Australia’s welfare series no. 13. Canberra, Australia: AIHW. https://www.aihw.gov.au/getmedia/088848dc-906d-4a8b-aa09-79df0f943984/aihw-aus-214-aw17.pdf.aspx?inline=true. Accessed September 24, 2019.

[22] Jamieson LM, Roberts-Thomson KF Indigenous children and receipt of hospital dental care in Australia. Int J Paediatr Dent. 2006;16(5):327-334. doi:10.1111/j.1365-263X.2006.00749.x 16879329

