# Supplementary material for: Follow-up of Intervention to Prevent Dental Caries Among Indigenous Children in Australia: A Secondary Analysis of a Randomized Clinical Trial
Source: JAMA Netw Open. 2019 Nov 27;2(11):e1915611. doi: 10.1001/jamanetworkopen.2019.15611 (PMC6902806; doi:10.1001/jamanetworkopen.2019.15611)
Supplement: Supplement. — eTable. Baseline Sample Characteristics by Follow-up and Loss-to-Follow-up at 5-Year Examinations [file jamanetwopen-2-e1915611-s001.pdf]

Supplementary Online Content

Jamieson L, Smithers L, Hedges J, et al. Follow-up of intervention to prevent dental caries among indigenous children in Australia: a secondary analysis of a randomized clinical trial. *JAMA Netw Open.* 2019;2(11):e1915611. doi:10.1001/jamanetworkopen.2019.15611

eTable. Baseline Sample Characteristics by Follow-up and Loss-to-Follow-up at 5-Year Examinations

This supplementary material has been provided by the authors to give readers additional information about their work.

eTable. Baseline Sample Characteristics by Follow-up and Loss-to-Follow-up at 5-Year Examinations

	**5 year follow-up**					**5 year loss to follow-up**			
	**N (%)**			***p-value**		**N (%)**			***p-value**
	Total (n=299)	Immediate Intervention (n=156)	Delayed Intervention (n=143)			Total (n=137)	Immediate Intervention (n=62)	Delayed Intervention (n=75)	
**Maternal age**				0.0260					0.5792
14-24	156 (52.2)	91 (58.3)	65 (45.5)			76 (55.5)	36 (58.1)	40 (53.3)	
25+	143 (47.8)	65 (41.7)	78 (54.6)			61 (44.5)	26 (41.9)	35 (54.7)	
**Education**				0.3503					0.3282
High school or less	203 (68.4)	109 (70.8)	94 (65.7)			111 (81.0)	48 (77.4)	63 (84.0)	
Trade or University	94 (31.7)	45 (29.2)	49 (34.3)			26 (19.0)	14 (22.6)	12 (16.0)	
**Income**				0.8617					0.9657
Job	47 (15.9)	25 (16.2)	22 (15.5)			13 (9.6)	6 (9.7)	7 (9.5)	
Centrelink	249 (84.1)	129 (83.8)	120 (84.5)			123 (90.4)	56 (90.3)	67 (90.5)	
**HCC status**				0.9902					0.7448
Yes	236 (80.8)	122 (80.8)	114 (80.9)			113 (85.6)	49 (84.5)	64 (86.5)	
No	56 (19.2)	29 (19.2)	27 (19.2)			19 (14.4)	9 (15.5)	10 (13.5)	
**Residential location**				0.1361					0.7829
Metropolitan	132 (44.8)	63 (40.7)	69 (49.3)			35 (25.7)	15 (24.6)	20 (26.7)	
Non- metropolitan	163 (55.2)	92 (59.3)	71 (50.7)			101 (74.3)	46 (75.4)	55 (73.3)	
**Usual reason visit dentist**				0.3109					0.0268
Problem	184 (63.7)	92 (60.9)	92 (66.7)			83 (63.8)	45 (73.8)	38 (55.1)	
Check-up	105 (36.3)	59 (39.1)	46 (33.3)			47 (36.2)	16 (26.2)	31 (44.9)	
**Brush yesterday**				0.6922					0.2102
Yes	217 (75.9)	116 (76.8)	101 (74.8)			96 (72.7)	39 (67.2)	57 (77.0)	
No	69 (24.1)	35 (23.2)	34 (25.2)			36 (27.3)	19 (32.8)	17 (23.0)	
**Self-rated oral health**				0.2597					0.0183
Excellent, very good or good	130 (43.5)	63 (40.4)	67 (46.8)			66 (48.2)	23 (37.1)	43 (57.3)	
Fair or poor	169 (56.5)	93 (59.6)	76 (53.2)			71 (51.8)	39 (62.9)	32 (42.7)	
**Self-rated general health**				0.2393					0.8450
Excellent, very good or good	272 (91.0)	139 (89.1)	133 (93.0)			119 (87.5)	53 (86.9)	66 (88.0)	
Fair or poor	27 (9.0)	17 (10.9)	10 (7.0)			17 (12.5)	8 (13.1)	9 (12.0)	

Note: * Chi-square test

©2019 Jamieson L et al. *JAMA Network Open*
